# Effects of Cocaine Self-Administration and Its Extinction on the Rat Brain Cannabinoid CB1 and CB2 Receptors

**DOI:** 10.1007/s12640-018-9910-6

**Published:** 2018-05-12

**Authors:** Beata Bystrowska, Małgorzata Frankowska, Irena Smaga, Lucyna Pomierny-Chamioło, Małgorzata Filip

**Affiliations:** 1 Department of Toxicology, Jagiellonian University Medical College, Medyczna 9, 30-688 Kraków, Poland; 2 Department of Drug Addiction Pharmacology, Institute of Pharmacology, Polish Academy of Sciences, Smętna 12, 31-343 Kraków, Poland; 3 Department of Internal Medicine, Jagiellonian University Medical College, Skawińska 8, 31-066 Kraków, Poland

**Keywords:** Cocaine self-administration, Cannabinoid receptors, Immunohistochemistry, Cocaine addiction

## Abstract

The aim of this study was to evaluate changes in the expression of cannabinoid type 1 (CB1) and 2 (CB2) receptor proteins in several brain regions in rats undergoing cocaine self-administration and extinction training. We used a triad-yoked procedure to distinguish between the motivational and pharmacological effects of cocaine. Using immunohistochemistry, we observed a significant decrease in CB1 receptor expression in the prefrontal cortex, dorsal striatum, and the basolateral and basomedial amygdala following cocaine (0.5 mg/kg/infusion) self-administration. Increased CB1 receptor expression in the ventral tegmental area in rats with previous cocaine exposure was also found. Following cocaine abstinence after 10 days of extinction training, we detected increases in the expression of CB1 receptors in the substantia nigra in both cocaine groups and in the subregions of the amygdala for only the yoked cocaine controls, while any method of cocaine exposure resulted in a decrease in CB2 receptor expression in the prefrontal cortex (*p* < 0.01), nucleus accumbens (*p* < 0.01), and medial globus pallidus (*p* < 0.01). Our findings further support the idea that the eCB system and CB1 receptors are involved in cocaine-reinforced behaviors. Moreover, we detected a cocaine-evoked adaptation in CB2 receptors in the amygdala, prefrontal cortex, and globus pallidus.

## Introduction

Cocaine is one of the most psychoactive drugs that leads to substance use disorder. The principal mechanism of action of cocaine is via monoamine (dopamine, noradrenaline, and serotonin) reuptake inhibition, which in turn enhances the synaptic concentration of these neurotransmitters (Jastrzębska et al. 2015; Nestler 2004; Wydra et al. 2013). Recently, it was discovered that cocaine may exert direct and/or indirect allosteric enhancing effects at dopamine (DA) D2 receptors (Ferraro et al. 2010), while cocaine use disorder is associated with a reduced density of striatal DA D2 receptor expression in the striatum (Blum et al. 2012). Interestingly, in rat striatal slices, cocaine—via D2-like receptor activation—and the D2-like receptor agonist quinpirole enhance the synthesis of the endocannabinoid anandamide and inhibit its degradation (Centonze et al. 2004), while in vivo, quinpirole stimulates the striatal release of anandamide in rats (Giuffrida et al. 1999). Functional crosstalk between D2 receptors and the endocannabinoid system also exists at the intracellular pathway level (Alonso et al. 1999; André and Gonthier 2010; Chiang et al. 2013; Fuxe at al. 2010).

Apart from anandamide, the endocannabinoid system includes another endogenous ligand (2-arachidonoylglycerol), enzymes responsible for the synthesis and degradation of endocannabinoids and G protein-coupled cannabinoid receptors, namely CB1 and CB2. The CB1 receptors are localized mainly in brain neurons, and their distribution depends on the structure in which they are found: the highest densities of CB1 receptors are found in the basal ganglia, hippocampus, and cerebellum (Herkenham et al. 1990, 1991; Thomas et al. 1992). CB1 receptors are also present in glutamatergic and GABAergic nerve endings (Kano et al. 2009; Wilson and Nicoll 2002).

CB2 receptors were initially recognized only in the immune system, however, although their presence has now been demonstrated in the brain as well (Engeli 2012; Gong et al. 2006; Onaivi et al. 2006; Van Sickle et al. 2005; Viscomi et al. 2009). In the brain, CB2 receptor transcripts and/or proteins have been detected in the retina (Lu et al. 2000), cortex, cerebral cortex (Ashton et al. 2006), hippocampus, amygdala, striatum, and brainstem (Van Sickle et al. 2005). In the above brain areas, the cellular localization of CB2 receptors is linked to the presence of microglia (Malfitano et al. 2014); however, but a recent study has also shown the expression of these receptors in DA neurons in the ventral tegmental area (Zhang et al. 2014; Zhang et al. 2016).

There are several reports supporting the control of the endocannabinoid system over cocaine use disorder, although the data are conflicting. At the behavioral level, the lack of CB1 receptors in mice (Soria et al. 2005) or pretreatment with a CB1 receptor agonist in rats (Fattore et al. 1999) reduces cocaine self-administration. Other pharmacological analyses using CB1 receptor antagonists do not reveal their effects on cocaine-reinforcing properties in rodents (Adamczyk et al. 2012b; Cossu et al. 2001; Lesscher et al. 2005), while the brain’s CB2 receptors were found to modulate the rewarding effect of cocaine (Xi et al. 2011; Zhang et al. 2014). The other discrepant data show that CB1 and CB2 receptor blockade attenuates the reinstatement of the drug and cue-associated cocaine-seeking behavior (Filip et al. 2006; Xi et al. 2006; Adamczyk et al. 2012b), while both stimulation and blockade of CB1 receptors play an important role in drug- as well as in cue-induced reinstatement of cocaine-seeking behavior (Fattore et al. 2007) in rats. A recent separate study by McReynolds et al. (2016) demonstrates that CB1 receptor antagonism blocks stress-potentiated reinstatement of cocaine seeking in rats (McReynolds et al. 2016).

Using autoradiographic procedures, it has been shown that either cocaine self-administration (Adamczyk et al. 2012a) or passive drug injections (Gonzalez et al. 2002; Centonze et al. 2004) evoke neuroadaptive changes in CB1 receptor density, which depend on the brain structure examined and last until drug abstinence occurs. On the other hand, García-Cabrerizo and García-Fuster (2016) with using Western blot analyses showed opposite and transient regulation of CB1 (increases) and CB2 (decreases) receptors in the prefrontal cortex of rats treated with cocaine during early adolescence (García-Cabrerizo and García-Fuster 2016). In cocaine addicts as well as in chronic cocaine-treated rodents, a reduction in CB1 receptor protein (but not in CB2 receptor protein) in the prefrontal cortex was demonstrated (Álvaro-Bartolomé and García-Sevilla 2013). Our findings related to the eCB brain levels during cocaine self-administration, yoked cocaine delivery, and cocaine withdrawal demonstrate significant changes in brain tissue endocannabinoid (eCB) levels and further support the significance of the eCB system in the reinforcement and extinction of positively reinforced behaviors (Bystrowska et al. 2014).

In view of these data, we used a triad-yoked procedure to distinguish between the motivational and pharmacological effects of cocaine, plus to evaluate the changes in the expression of CB1 and CB2 receptors in different rat brain structures in relation to the administration of cocaine in cellular membranes.

## Materials and Methods

### Animals

Male Wistar rats (280–300 g, *N* = 46) were delivered by a licensed breeder (Charles River, Germany) and were housed individually in standard plastic rodent cages in an animal colony room maintained at 20 ± 1 °C and at 40–50% humidity under a 12-h light-dark cycle (lights on at 06:00). The animals had free access to standard animal food and water during the 7-day habituation period. All of the experiments were conducted during the light phase of the light-dark cycle (between 08:00 and 15:00) and were carried out in accordance with the National Institutes of Health Guide for the Care and Use of Laboratory Animals and with the approval of the Bioethics Commission in compliance with Polish Law (21 August 1997). The animals were experimentally naive.

### Drugs

Cocaine hydrochloride (Sigma-Aldrich, St. Louis, USA) was dissolved in sterile 0.9% NaCl and given *iv* (0.1 ml/infusion).

### Behavioral Procedures

#### Cocaine Self-Administration and Extinction Training

Rats were trained to press the lever of standard operant conditioning chambers (Med-Associates, USA) under an increasing schedule of water reinforcement at a fixed ratio of 1 to 5. Two days following the “lever-press” training under FR5 conditions and with free access to water, the rats were chronically implanted with a silastic catheter in the external right jugular vein, as described previously (Frankowska et al. 2010). The catheters were flushed every day with 0.1 ml of a saline solution containing heparin (70 U/ml, Biochemie GmbH, Austria) and 0.1 ml of a cefazolin solution (10 mg/ml; Biochemie GmbH, Austria). There were no problems with catheter patency.

After a 7- to 8-day recovery period, all of the animals were water-deprived for 18 h and trained to press the lever at an FR 5 schedule of water reinforcement over a 2-h session for 14 days. The subjects were then given access to cocaine during 2-h daily sessions performed 6 days/week (maintenance), and during that time, they were given water ad libitum. The house lights were illuminated throughout each session. Each completion of five presses on the “active” lever complex (a FR 5 schedule) resulted in a 5-s infusion of cocaine (0.5 mg/kg per 0.1 ml) and a 5-s presentation of a stimulus complex (the activation of a white stimulus light directly above the active lever and a tone generator set at 2000 Hz and 15 dB above the ambient noise levels). Following each injection, there was a 20-s time-out period during which responses were recorded but no programmed consequences occurred. Responses involving the “inactive” lever resulted in no delivery of cocaine. The acquisition of the conditioned operant response until the subjects met the following criteria: active lever presses over an average of three consecutive days and with a standard deviation within those 3 days of < 10% of the average number of lever presses. This criterion was selected based on our prior experiments (Filip et al. 2006). After the 14th (2-h) self-administration session, the animals were decapitated (group 1; *N* = 6).

Separate groups of animals trained to self-administer cocaine (0.5 mg/kg/inf) for 14 days were exposed to the extinction training. During the extinction sessions, the subjects had 2-h daily training sessions with no cocaine delivery (saline was substituted for cocaine) or presentation of the conditioning stimulus. Once they reached the extinction criteria (10 extinction days with the last 3 days in which active lever responses were below 10% of the level observed during the maintenance period), on the 10th day of extinction, the animals were sacrificed immediately following the last (2-h) experimental session (group 2; *N* = 7).

#### Yoked Self-Administration Procedure

The rats were tested simultaneously in groups of three, with two rats serving as “yoked” controls that received an injection of saline (*N* = 6–7) or cocaine (*N* = 6–7) which was not contingent on their response and occurred each time a response-contingent injection of 0.5 mg/kg of cocaine was self-administered by the paired rat. For yoked saline and yoked cocaine controls, the 5-s presentation of a stimulus complex (see above) was present during the infusion followed by the 20-s time-out period. Unlike self-administering rats, lever pressing by the yoked rats was recorded but had no programmed consequences (Frankowska et al. 2008).

### Immunohistochemistry Procedures

**Fig. 1:**
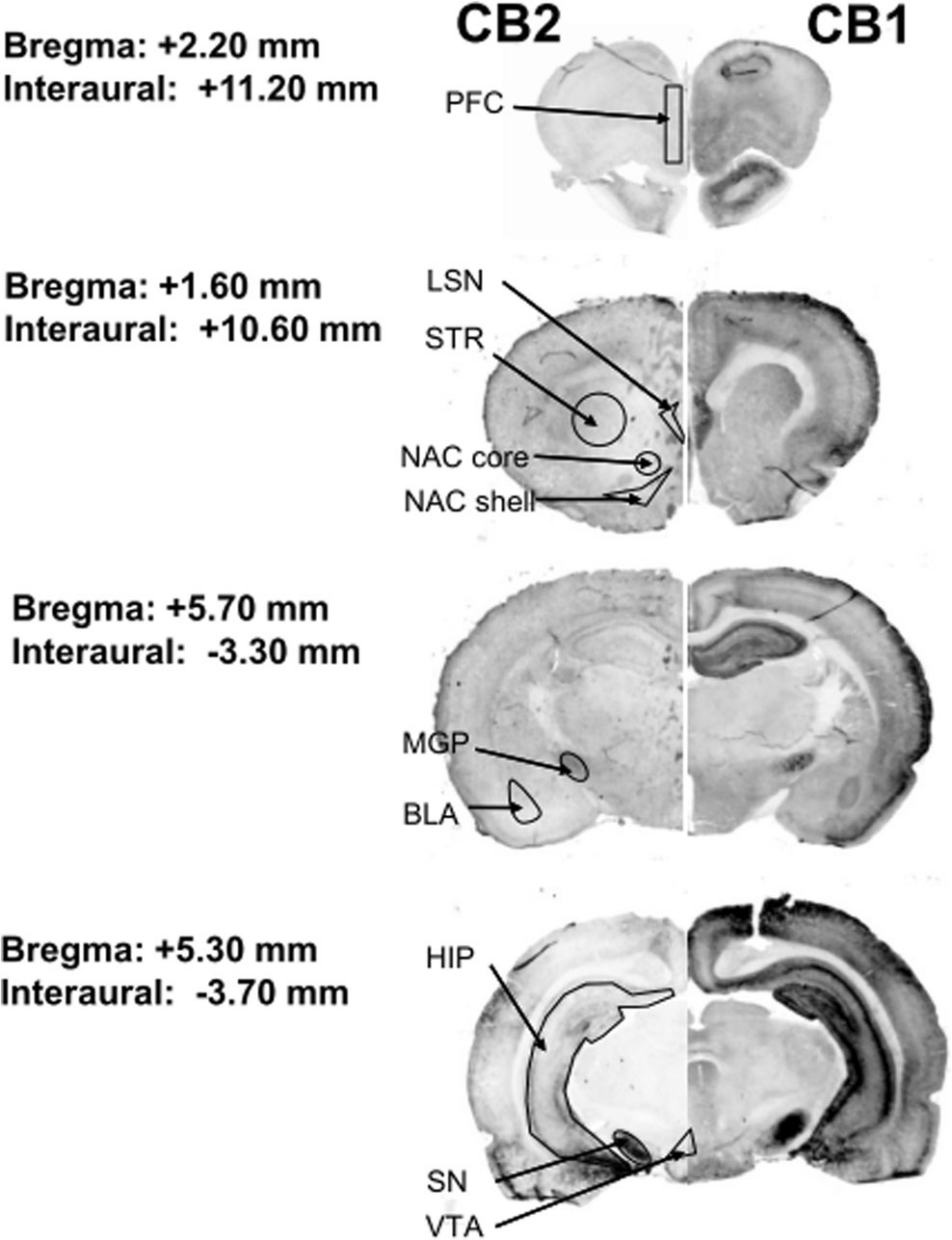
Representative coronal sections of rat brain immunostaining for CB1 and CB2 receptors after 14 days of passive saline administration (control group). The panels are gray scale images of individual antibodies. BLA basolateral and basomedial amygdala, DST dorsal striatum, HIP hippocampus, LSN lateral septal nucleus, MGP medial globus pallidus, NAc core nucleus accumbens core, NAc shell nucleus accumbens shell, PFC prefrontal cortex, SN substantia nigra, VTA ventral tegmental area

**Fig. 2:**
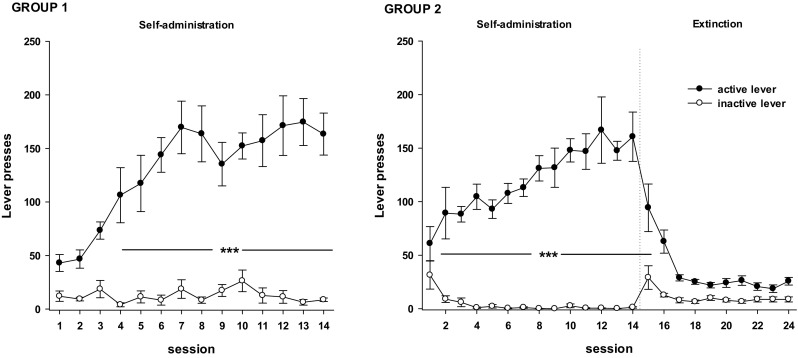
The number of “active” and “inactive” lever presses in rats underwent cocaine (0.5 mg/kg/infusion) self-administration (left panels; group 1) or 10-daily cocaine abstinence with extinction training (right panels, group 2). Data are presented as the mean ± SEM. ***p < 0.0001 versus “inactive” lever

Immediately after the experimental sessions, the rats were injected with pentobarbital and perfused intracardially with a solution of 4% paraformaldehyde in 100 mM phosphate-buffered saline (PBS), pH = 7.4. The brains were excised and postfixed in the same fixative for 12 h. The tissue was permeated with 10% *w*/*v* sucrose for 7 days and then removed to a 30% *w*/*v* sucrose solution in PBS at 4–8 °C for a minimum of 48 h. The brains were fully frozen on dry ice, cut into 12-μm coronal sections on a cryostat (Leica Microsystems, Nussloch, Germany), and kept at − 20 °C until they were processed for immunohistochemical analysis. Later, the rat brain sections were rinsed in 100 mM PBS (pH = 7.4) and in PBS containing 0.1% Triton X-100 for 30 min at room temperature (RT). They were then rinsed with Odyssey Blocked Buffer (OBB) for 1 h at room temperature. The tissue sections were incubated overnight at 4 °C with the following purified primary antibodies in OBB containing 0.1% Tween 20: rabbit anti-CB1 (1:200 dilution, Abcam, ab23703) and goat anti-CB2 (1:200 dilution, Santa-Cruz, sc10076). Following four 5-min washes with PBS containing 0.1% Tween 20, the tissue sections were incubated for 1 h at room temperature with goat anti-rabbit (IRDye® 680CW) and donkey anti-goat (IRDye® 800CW) secondary antibodies (Li-COR Biosciences, Cambridge, UK) at a dilution of 1:2000. Fluorescence was detected using the Odyssey® Infrared Imaging System (21-μm resolution, 1-mm offset at the highest resolution). The integrated intensities were determined with the associated Odyssey software. Each section was prescanned at different intensity settings on the Odyssey Classic Infrared Imaging System. Channel sensitivity was optimized for each set of stained sections, and channel intensity varied from 3 to 5. The latter allows detection of nonspecific background signals from the sample and permits gross localization of the cerebral tissue. The relative location of the slices and the identification of brain regions were determined via comparisons to the images in The Rat Brain Atlas (1998). Schematic illustrations of rat brain structures are shown in Fig. 1, and representative coronal sections of rat brains immunostained for the CB1 and CB2 receptors after 14 days of passive saline administration are shown in Fig. 2.

### Data Analysis

Animals that did not meet the training criteria for performing cocaine self-administration (*N* = 2) and extinction (*N* = 1) or those showing incorrect perfusion (*N* = 3) were excluded from the data analysis. All data are expressed as the means (± SEM). Behavioral data were analyzed using a two-way ANOVA for repeated measures followed by post hoc Newman-Keuls tests. Immunohistochemical data were analyzed using a one-way ANOVA, and appropriate post hoc comparisons were performed using Newman-Keuls tests. The results with a *p* value < 0.05 were considered statistically significant.

## Results

### Behavioral Studies

After the self-administration sessions, animals in the two experimental groups showed stable lever-pressing rates during the last three self-administration days, with less than a 10% difference in their daily intake of cocaine (Fig. 2). The mean number of cocaine infusions per day during the last three self-administration days varied from 26 to 29. During 14 experimental sessions, the animals received from 158.0 to 176.4 mg/kg of cocaine each. Rats trained to self-administer cocaine (group 1) pressed on the active lever significantly more frequently than they pressed on the inactive lever from the 4th to the 14th experimental session, as assessed by the lever × day session interaction (*F*_(13,130)_ = 4.65, *p* < 0.001).

Following 14 days of cocaine self-administration, the extinction training started for a separate group of animals. In this phase, neither drug nor drug-paired stimuli were given in response to lever pressing, which resulted in a gradual decrease in active lever presses. In the group of rats decapitated on the 10th day of extinction training, the animals pressed on the active lever more frequently than they pressed on the inactive lever from the 2nd cocaine self-administration session until the 1st day of extinction training (*F*_(23,276)_ = 11.52, *p* < 0.001).

In the yoked saline and cocaine groups, the animals received saline or the same amount of cocaine at the same time as the animals that learned to self-inject cocaine, respectively. In the yoked cocaine groups, the total number of active lever presses did not differ from the inactive lever as shown by a two-way ANOVA for repeated measures (14 days with yoked cocaine delivery (*F*_(13,130)_ = 1.02); 14 days with yoked cocaine delivery and 10 days of cocaine abstinence (*F*_(23,276)_ = 0.98)); however, some increases in the active lever presses were seen during the initial three to four experimental sessions due to associate learning that was occurring in the yoked control groups. In the yoked saline groups (14 days with yoked saline delivery as well as 24 days with yoked saline delivery), the difference in pressing the active versus the inactive lever failed to reach significance (*F*_(13,130)_ = 0.37 and *F*_(23,276)_ = 0.29, respectively*)*.

### Expression of the CB1 and CB2 Receptor Protein

**Fig. 3:**
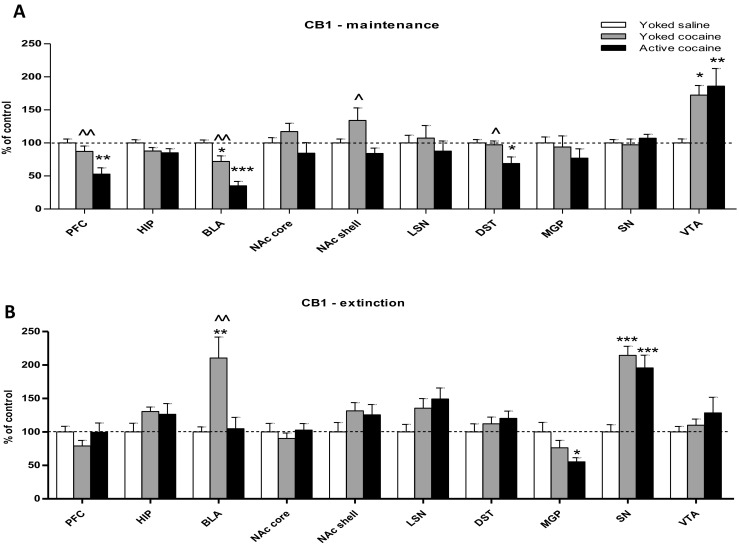
Immunohistochemical analyses for the CB1 receptor expression in the rat brain structures following cocaine (0.5 mg/kg/infusion) self-administration (**a**) and 10-daily cocaine abstinence with extinction training (**b**). Active cocaine—rats self-administering cocaine; Yoked cocaine—rats received passively cocaine; Yoked saline—rats received passively saline. For more explanation see Fig. 1. *N* = 6 rats/group for cocaine self-administration; *N* = 7 rats/group for cocaine abstinence. *p < 0.05, **p < 0.01, ***p < 0.001 vs Yoked saline; ^*p* < 0.05, ^^*p* < 0.01, ^^^*p* < 0.001 vs Active cocaine (Newman-Keuls test)

As shown in Figs. 3a and 5, cocaine self-administration in rats resulted in a decrease in the expression CB1 receptor proteins in the prefrontal cortex (*F*_(2,15)_ = 9.239; *p* < 0.01), basolateral and basomedial amygdala (*F*_(2,15)_ = 22.906; *p* < 0.001) and dorsal striatum (*F*_(2,15)_ = 5.497; *p* < 0.05), whereas in the nucleus accumbens shell of the yoked cocaine controls, there was a significant increase in CB1 receptor expression (*F*_(2,15)_ = 4.344; *p* < 0.05). In both cocaine groups, we found enhanced (> 50%) tegmental CB1 receptor protein expression (*F*_(2,15)_ = 6.668; *p* < 0.01). There were no changes in CB1 receptor protein expression in the lateral septal nuclei (*F*_(2,15)_ = 0.413), nucleus accumbens core (*F*_(2,15)_ = 1.673), medial globus pallidus (*F*_(2,15)_ = 0.756), hippocampus (*F*_(2,15)_ = 2.074), and substantia nigra (*F*_(2,15)_ = 0.574), as revealed by a one-way ANOVA (for all, *p* > 0.05).

The 10-daily cocaine abstinence period with extinction training sessions in rats self-administered cocaine evoked the statistically significant reduction in CB1 receptor expression in the medial globus pallidus (*F*_(2,18)_ = 4.097; *p* < 0.05) (Figs. 3b and 5). However, there was a significant increase in CB1 receptor expression in the substantia nigra (*F*_(2,18)_ = 17.026; *p* < 0.001) in both cocaine groups and in the basolateral and basomedial amygdala (*F*_(2,18)_ = 8.927; *p* < 0.01) in the cocaine-yoked controls. Cocaine abstinence did not change CB1 receptor expression in the prefrontal cortex (*F*_(2,18)_ = 1.279), hippocampus (*F*_(2,18)_ = 1.489), nucleus accumbens shell (*F*_(2,18)_ = 1.428), nucleus accumbens core (*F*_(2,18)_ = 0.397), lateral septal nuclei (*F*_(2,18)_ = 3.231), dorsal striatum (*F*_(2,18)_ = 0.845), and ventral tegmental area (*F*_(2,18)_ = 0.900; *p* > 0.05), as assessed by a one-way ANOVA (*p* > 0.05).

**Fig. 4:**
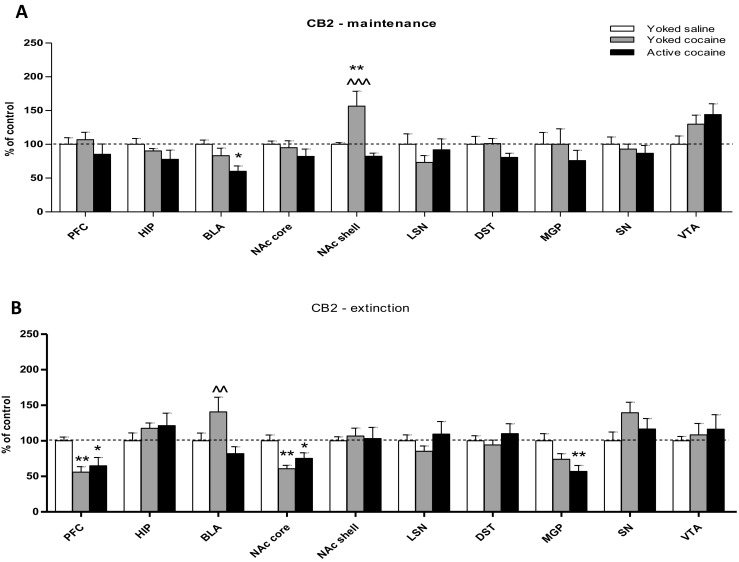
Immunohistochemical analyses for the CB2 receptor expression in the rat brain structures following cocaine (0.5 mg/kg/infusion) self-administration (**a**) and 10-daily cocaine abstinence with extinction training (**b**). Active cocaine—rats self-administering cocaine; Yoked cocaine—rats received passively cocaine; Yoked saline—rats received passively saline. For more explanation see Fig. 1. *N* = 6 rats/group for cocaine self-administration; *N* = 7 rats/group for cocaine abstinence. *p < 0.05, **p < 0.01, ***p < 0.001 vs Yoked saline; ^^*p* < 0.01 vs Active cocaine (Newman-Keuls test)

**Fig. 5:**
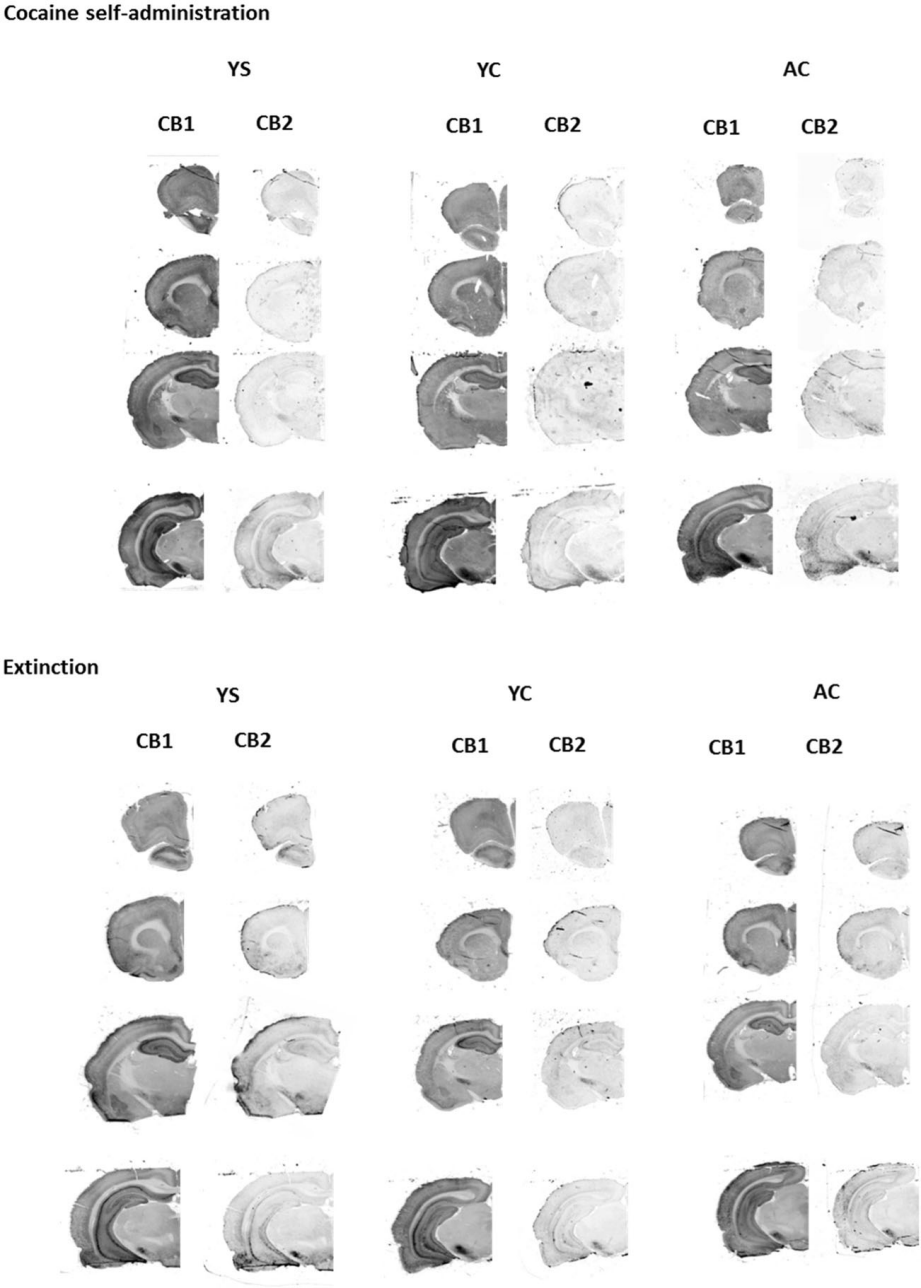
Representative coronal sections of rat brain immunostaining for CB1 and CB2 receptors after cocaine self-administration and 10-daily cocaine abstinence with extinction training. The panels are gray scale images of individual antibodies. Active cocaine (AC)—rats self-administering cocaine; Yoked cocaine (YC)—rats received passively cocaine; Yoked saline (YS)—rats received passively saline

Cocaine self-administration produced a statistically significant decrease in CB2 receptor protein expression in the basolateral and basomedial amygdala (*F*_(2,15)_ = 5.489; *p* < 0.05). As demonstrated for the nucleus accumbens shell, yoked cocaine administration increased the expression of CB2 proteins (*F*_(2,15)_ = 8.778; *p* < 0.01). There were no changes in either cocaine group in CB2 receptor expression (*p* > 0.05) in the prefrontal cortex (*F*_(2,15)_ = 0.816), hippocampus (*F*_(2,15)_ = 1.374), nucleus accumbens core (*F*_(2,15)_ = 1.022), lateral septal nuclei (*F*_(2,15)_ = 0.932), dorsal striatum (*F*_(2,15)_ = 1.701), medial globus pallidus (*F*_(2,15)_ = 0.542), substantia nigra (*F*_(2,15)_ = 0.429), and VTA (*F*_(2,15)_ = 2.601) (Figs. 4a and 5), as shown by a one-way ANOVA.

Cocaine abstinence in both cocaine groups altered CB2 receptor protein expression, with significant (> 40%) decreases observed in the prefrontal cortex (*F*_(2,18)_ = 7.305; *p* < 0.01), nucleus accumbens core (*F*_(2,18)_ = 7.638; *p* < 0.01), and medial globus pallidus (*F*_(2,18)_ = 6.051; *p* < 0.01). There were no changes (*p* > 0.05; one-way ANOVA) in CB2 receptor protein expression in the hippocampus (*F*_(2,18)_ = 0.809), basolateral and basomedial amygdala (*F*_(2,18)_ = 4.206), nucleus accumbens shell (*F*_(2,18)_ = 0.087), lateral septal nuclei (*F*_(2,18)_ = 1.014), dorsal striatum (*F*_(2,18)_ = 0.647), substantia nigra (*F*_(2,18)_ = 1.989), and ventral tegmental area (*F*_(2,18)_ = 0.282) (Figs. 4b and 5).

## Discussion

A growing number of behavioral studies support the important role of eCB signaling in substance use disorder, including cocaine addiction (Mereu et al. 2015; Onaivi 2008; Serrano and Parsons 2011; Adamczyk et al. 2012b; Fattore et al. 1999; Filip et al. 2006). The present results extend our previous neurochemical observations (Adamczyk et al. 2012a; Bystrowska et al. 2014) and behavioral (Adamczyk et al. 2012b) observations as well as further outline the significance of the eCB system and its receptors in the reinforcing and motivational effects of cocaine.

We report here that cocaine self-administration for 14 days in 2-h daily sessions in rats causes a significant reduction in CB1 receptor expression in the prefrontal cortex, basolateral and basomedial amygdala, and dorsal striatum. These changes did not appear (except in the amygdala) after repeated passive iv cocaine exposure, indicating that these effects may be linked to the motivational, associate learning and/or locomotor aspects of cocaine intake. Immunohistochemical staining also revealed a large increase (> 60%) in CB1 receptor expression in the ventral tegmental area as a result of the pharmacological aspects of cocaine use, as revealed after both voluntary or passive psychostimulant intake, and/or the Pavlovian associations between drug delivery and the conditioned stimulus (the tone+light associated with cocaine infusions).

Furthermore, the above changes in CB1 receptor expression disappeared after extinction training, when a 50% reduction in CB1 receptor expression in rats with a history of cocaine self-administration and a > 100% increase in receptor protein expression after previous active or passive cocaine injections were noted. Interestingly, it was only in the cocaine-yoked controls that cocaine abstinence evoked a significant increase in CB1 receptor expression in the rat amygdala.

The present data extend our previous observations on brain tissue eCB levels using the same behavioral protocol in rats (Bystrowska et al. 2014). Moreover, our neurochemical ex vivo results support previously obtained direct in vivo evidence of cocaine-induced alterations in the nucleus accumbens of mice as well as behavioral data on cocaine self-administration following intra-accumbal injections of a CB1 receptor antagonist (Caillé et al. 2007). However, other pharmacological and genetic approaches have not supported the idea that cocaine-induced reward behavior is sensitive to this type of CB1 receptor manipulation (Chaperon et al. 1998; Martin et al. 2000; Tanda et al. 2000; Adamczyk et al. 2012a; Fattore et al. 1999; Filip et al. 2006). Although it is difficult to explain the above differences, a recent paper by Martín-García et al. (2016) shows that CB1 receptors—localized mainly to glutamatergic and GABAergic neuronal systems in the brain—are involved in different aspects of cocaine addiction (Martín-García et al. 2016). Using mutant mice with CB1 receptors deleted from their cortical glutamatergic neurons (i.e., in the cortex, hippocampus, and cortical portions of the amygdala) and from the GABAergic neurons in their forebrain (i.e., in the whole striatum, thalamus, and hypothalamus), these authors found that CB1 receptors in cortical glutamatergic neurons control associative learning processes, whereas CB1 receptors in GABAergic neurons in the forebrain control the sensitivity to cocaine. Based on these observations, the reductions in CB1 receptor expression in cortical and amygdaloidal structures presented in this paper may reflect the cocaine-induced facilitation of associative learning, which is a key process controlling cocaine use (Hogarth et al. 2013; Voon et al. 2014), and the perception of the drug’s importance, respectively. Regarding the striatum, it plays a role in skill learning, instrumental conditioning, habit formation, and the transition from voluntary to habitual drug seeking (Everitt and Robbins 2013) and CB1 receptor deletion from olfactory-dorsal striatal neurons prevents the shift from goal-directed to habitual action control (Gremel et al. 2016). It should also be added that between the basolateral amygdala (i.e., a primary locus in mediating the associations between the input of a drug stimulus and the subsequent drug-seeking behavior) and the nucleus accumbens core/dorsal striatum, there are direct glutamatergic interactions that control cocaine-seeking behavior in rats (Di Ciano and Everitt 2004). Our data suggest that drugs modulating eCB transmission at CB1 receptors may be useful in future treatments to reduce the incentive value of the cocaine-associated reward or to prevent cocaine addiction through the blockade of memory reconsolidation.

In contrast to the above noted decreases in expression linked (mainly) to voluntary cocaine intake, the increases observed in tegmental CB1 receptor expression seem to be a pharmacological effect of cocaine delivery. It should also be noted that cocaine delivery in self-administering rats and yoked drug controls are linked with a conditioned stimulus, and the ventral tegmental area plays a significant role in learning and habit formation (Barker and Rebec 2016). In fact, Wang et al. (2015) recently demonstrated the control of tegmental CB1 receptors over cocaine-induced synaptic plasticity and associative learning (Wang et al. 2015). The latter process has also been found to occur in rats given a passive drug injection in conditioned place preference trials and were found to be linked with the cocaine-induced enhancement of extrasynaptic 2-AG that acts via presynaptic CB1 receptor activation to inhibit GABAergic inputs to GABAB receptors localized at the dopamine neurons. Further in vivo studies are required to determine if tegmental CB1 receptors are a critical biomarker involved in both acquiring and retrieving cocaine-associated memories.

The nucleus accumbens is a gateway for emotional, motivational, or locomotor responses. Although the level of expression of CB1 receptors in this brain structure is low, they are localized to afferents to the nucleus accumbens and to accumbal fast-spiking interneurons (Winters et al. 2012). Furthermore, CB1 receptor-expressing fast-spiking interneurons form GABAergic synapses with adjacent medium spiny neurons, which generate a feed-forward inhibition of accumbal output. As shown previously, cocaine self-administration and noncontingent exposure to cocaine reduce the membrane excitability of medium spiny neurons (Ishikawa et al. 2009; Mu et al. 2010), and cocaine acts on CB1 receptors localized to fast-spiking interneurons to further increase their inhibitory influence over medium spiny neurons (Winters et al. 2012). As ventral striatopallidal GABA neurons (Trifilieff et al. 2011) represent an antireward system modulating the glutamate drive to the prefrontal cortex over the ventral pallidum and the dorsomedial thalamic nucleus (Fuxe et al. 2008), CB1 receptors in the nucleus accumbens may be neuronal targets for cocaine-induced neuroadaptations.

As mentioned above, the pattern of CB1 receptor expression was different during the cocaine-free period. Thus, 10 days of cocaine abstinence that included extinction training caused bi-phasic changes in the pattern of CB1 receptor expression, with a significant decrease in expression in the medial globus pallidus and a potent increase in expression in the substantia nigra. The globus pallidus is responsible, in addition to its other roles, for controlling cocaine-seeking behaviors, and the reduction in the expression of CB1 receptors on striatopallidal axons in the globus pallidus may disturb the presynaptic inhibition of GABA neurotransmission and globus pallidus neurons, which in turn could allow for multiphasic motor effects such as cocaine seeking (Engler 2005; Gonzalez et al. 2009). This hypothesis needs to be confirmed using functional assays. On the other hand, we demonstrate that cocaine abstinence in yoked cocaine controls resulted in a significant enhancement of CB1 receptor expression in the basolateral amygdala. There are literature data showing that basolateral amygdala integrates and processes emotions such as fear and anxiety that promote survival by warning of potential danger (Sharp 2017) and that basolateral eCB system may be involved in regulating the stress responses, fear, and anxiety (Gorzalka et al. 2008; Hill and Gorzalka 2004; Hill et al. 2008; Nasehi et al. 2018). As yoked cocaine can evoke a stress response in rats, a rise in CB1 receptor expression in this region might reflect disrupted EC signaling with an inability to adapt to stress.

This study also suggests that the CB1-receptor-mediated plasticity of dopamine neurons in the substantia nigra can contribute to cocaine-induced modulation at a cellular level. Supporting this idea, previous research shows that the substantia nigra and its dopamine neurons are engaged in the rewarding aspects of cocaine use (Ilango et al. 2014; Ramayya et al. 2014; Rossi et al. 2013; Wise 2009). On the other hand, a rise in CB1 protein expression in the substantia nigra of yoked cocaine animals which were exposed to cues, and—despite the possibility to associate the drug cue to the operant response—they formed a Pavlovian association between the cue presentation and cocaine effects which were messed during animals’ exposure to experimental chambers in the absence of cocaine and its cue. Since the findings also demonstrate a critical function of the nigrostriatal dopamine system in controlling actions as the dopamine neurons in the substantia nigra were excited during a Pavlovian procedure with appetitive and aversive outcomes (Matsumoto and Hikosaka 2009), it may be considered here that the nigral CB1 receptor may signal—through the midbrain dopaminergic neurons—the occurrence of unpredicted reward, which is used in appetitive learning to reinforce existing actions (Redgrave and Gurney 2006; Schultz 2007).

In the next set of experiments, we showed the significance of CB2 receptors in cocaine-induced behaviors. Compared to the levels in peripheral organs (e.g., the spleen), the brain CB2 mRNA and protein levels are very low, and the molecules are localized mainly at astrocytes (Aracil-Fernández et al. 2012; Baek et al. 2013; Ashton et al. 2006; Zhang et al. 2014). Recent findings have demonstrated the wide distribution of CB2 receptors in rodent brains in tegmental dopaminergic and nondopaminergic neurons (Zhang et al. 2015; Aracil-Fernández et al. 2012; Zhang et al. 2014; Zhang et al. 2016) and in hippocampal pyramidal neurons (Kim and Li 2015; Li and Kim 2015) and have shown the presence of striatal GABAergic neurons in nonhuman primates (Lanciego et al. 2011; Sierra et al. 2015).

In the present paper, we detected a significant reduction of CB2 receptor expression in the basolateral and basomedial amygdala which may be linked to the motivational aspects of cocaine intake. There was a potent increase in the expression of this receptor protein in the accumbal shell in the yoked cocaine controls and a nonsignificant increase in CB2 receptor expression in the ventral tegmental area in both cocaine groups. Until now, a single report indicated that cocaine self-administration upregulates CB2 mRNA expression in the prefrontal cortex and striatum and in tegmental dopaminergic neurons (Zhang et al. 2016). Whether our and the later authors’ findings speak for the engagement of brain CB2 receptors in cocaine reward or locomotor actions, further functional studies are required. So far, the enhancement of CB2 receptor function via pharmacological or genetic (Ignatowska-Jankowska et al. 2013; Xi et al. 2011; Zhang et al. 2014; Zhang et al. 2015) manipulations inhibits cocaine-associated locomotion, self-administration, and conditioned place preference in rodents (Aracil-Fernández et al. 2012).

This is also the first report showing a cocaine-induced change in CB2 receptor expression in the drug-free period. In fact, there was significant reduction in CB2 receptor expression in prefrontal cortex, nucleus accumbens core, and globus pallidus in both cocaine-experienced groups of animals. First, we postulated that the observed molecular processes of the eCB signaling at CB2 cannot be linked directly to messed contextual encoding of extinction or enhanced extinction consolidation, as such effects should be observed only in animals with a history of cocaine self-administration. On the other hand, the reduction in CB2 receptor signaling in the prefrontal cortex may reflect diminished emotional recognition that occurred in cocaine abstinent rats. In fact, the overexpression of CB2 receptors reduces anxiogenic behaviors and modifies the stress response (Ignatowska-Jankowska et al. 2013; Xi et al. 2011; Zhang et al. 2014, Zhang et al. 2015), and brain CB2 receptors are also functionally involved in depressive behaviors (García-Gutiérrez et al. 2010; García-Gutiérrez and Manzanares 2011). Changes in CB2 receptor expression in the amygdala—a brain structure responsible for detecting fear and preparing for emergency events—may also be a marker of mood disturbances that occurred in yoked cocaine control rats. In fact, stress is an inherent complication for yoked animals, and the aversive procedure can reduce the motivational aspect of cocaine (Twining et al. 2009) and enhances the stress hormone (corticosterone) levels. However, the significance of CB2 receptor alterations in cocaine abstinence needs to be determined in functional studies with using pharmacological tools.

## Conclusion

As shown in this study, alterations in the expression of CB1 and CB2 proteins in rat brain structures indicate their importance to cocaine-induced reward and drug-seeking behaviors. Defining the functional role of a particular cannabinoid receptor in cocaine use disorder and determining whether the pharmacological modulation of this system may be an effective therapy for drug addiction requires further investigations.
